# Job satisfaction, professional competence, and self-efficacy: a multicenter cross-sectional study among registered nurses in Sweden and Norway

**DOI:** 10.1186/s12913-024-11177-8

**Published:** 2024-06-14

**Authors:** Stina Kallerhult Hermansson, Fredrik Norström, Yvonne Hilli, Jonas Rennemo Vaag, Karin Bölenius

**Affiliations:** 1 Department of Nursing, Umeå University, Umeå, 901 87 Sweden; 2 Department of Epidemiology and Global Health, Umeå University, Umeå, 901 87 Sweden; 3 Faculty of Nursing and Health Sciences, Nord University, Bodø, 8049 Norway

**Keywords:** Job satisfaction, Norway, Professional competence, Registered nurses, Self-efficacy, Sweden

## Abstract

**Background:**

Healthcare organizations worldwide face persistent challenges relating to turnover and intention to leave the nursing profession among registered nurses. Factors contributing to their retention and well-being at work include high job satisfaction, professional competence, and self-efficacy. Few multicenter studies have investigated these factors in relation to work experience in a Nordic context. Therefore, this study aimed to investigate job satisfaction, professional competence, and self-efficacy among registered nurses.

**Methods:**

This multicenter cross-sectional study survey was part of a larger overarching Swedish-Norwegian project, and was conducted among registered nurses (*n* = 1137) in September 2021. The participants worked in a variety of health care units, e.g., hospital units, primary health care, and home care. Data was subjected to descriptive and comparative statistical analysis; chi-square test, one-way between-groups analysis of variance (ANOVA) and Kruskal-Wallis test.

**Results:**

The findings show that job satisfaction is reported as lowest in registered nurses with medium-term work experience as compared to newly qualified and long-term work-experienced registered nurses. Professional competence and self-efficacy are reported as higher among registered nurses with long-term work experience as compared to those with medium-term work experience and newly qualified registered nurses. However, the participants reported their professional competence as highest in relation to the same factor – “Value-based nursing care” – regardless of their work experience.

**Conclusions and implications:**

This study underscores the need for continuous support and professional development for registered nurses throughout their careers. Proactive support for newly qualified nurses may improve job satisfaction as they progress to being registered nurses with medium-term work experience. Tailored interventions to address the distinct needs of both newly qualified and medium-term work-experienced registered nurses are crucial for nurturing a sustainable nursing workforce.

## Background

Healthcare organizations worldwide struggle with problems relating to high turnover rates among registered nurses (RNs) [1] and their intention to leave the profession [2, 3]. The gap between the supply of nurses and demand for them is increasing [4]. The gap is attributed to the global increase in demand for RNs, which corresponds with the streamlining of healthcare systems by many organizations and policymakers. These efforts aim to enhance efficiency and productivity, resulting in fewer employed RNs [1]. The reasons for retention challenges are complex and multifaceted [1–3]. The reduced number of RNs implies that those who opt to remain in the nursing profession be given more extensive responsibilities and an increased workload. This puts RNs at risk of stress and burnout, which impacts their well-being and job performance [5], along with the quality of patient care [1, 3]. Among other factors, the retention and well-being at work of RNs have been associated with high job satisfaction [3, 6], high self-reported professional competence [7], and strong self-efficacy [6]. At present, there is no consensus or agreement on how these factors differ in regard to work experience. Thus, in this study, we focused on how RNs self-reported these factors concerning their work experience.

Job satisfaction is traditionally defined as the enjoyable emotional state that arises when an individual’s work aligns with professional values and standards [8]. In a recent analysis, job satisfaction was described as the positive emotional response to work conditions that fulfil desired needs, based on the evaluation of the value or equity experienced in the work environment [9]. Factors such as education, motivation, commitment, support, collaboration, and leadership influence job satisfaction [9]. Although the findings regarding this are contradictory: one study found that higher age is correlated with higher job satisfaction and better performance [10], while another found a correlation between lower age and higher job satisfaction [11].

High job satisfaction and work engagement have also been associated with the general health and well-being of RNs [12], although it remains unclear whether job satisfaction and work engagement lead to improved health and well-being, or if better health and well-being enhance job satisfaction and work engagement. Job satisfaction is essential to experienced RNs intending to stay in the profession [13, 14]. It has been found that one in five RNs with five (or more) years of work experience intend to leave the profession [15], but a recent study found that RNs with at least five years of work experience reported higher job satisfaction than those who are just starting their careers [16].

Professional competence, comprising both knowledge and skills [17], is cultivated throughout an RN’s career, and forms the cornerstone of their ability to meet the demands of nursing practice [18]. The journey towards becoming secure as an RN entail gaining a profound understanding of the various situations they might encounter throughout their career. This journey is, according to Benner, characterized by distinct stages: novice, advanced beginner, competent, proficient, and expert [19]. Benner argues that it is crucial to understand the differences between experienced and novice nurses to facilitate the long-term and ongoing career development needed to manage the nursing practice’s complexity and responsibility [19]. A well-developed sense of competence is essential for instilling a profound sense of security among RNs. Furthermore, previous research indicates a positive correlation between work experience and self-assessed competence levels [20].

The self-efficacy of RNs plays a pivotal role in healthcare delivery, and has been linked to patient satisfaction [6]. High self-efficacy has been found to be related to prevention of burnout symptoms [21] and turnover intention among RNs [5, 21]. RNs with more experience have reported higher self-efficacy than their less experienced colleagues in previous studies [22, 23]. Self-efficacy, at its core, is an individual’s belief in their ability to execute the necessary actions to achieve desired outcomes [24]. It encompasses the confidence to navigate change [5] and belief in one’s competence to perform effectively under various circumstances—a measure of one’s self-perceived capability to tackle a task successfully [25].

The literature review presented in the background shows an interplay between work experience, job satisfaction, professional competence, and self-efficacy. It underscores the multifaceted nature of a RN’s journey toward becoming proficient, and highlights the importance of recognizing and nurturing these attributes in nursing practice. The healthcare landscape and working environments of RNs have significantly evolved and transformed over the past decade. This shift has necessitated a recent and comprehensive investigation regarding these critical factors in the Swedish-Norwegian context, in order to contribute to the overall knowledge base and for targeted interventions. To address this need, the present study aims to investigate job satisfaction, professional competence, and self-efficacy among registered nurses in Sweden and Norway, and to compare newly qualified nurses, medium-term work-experienced, and long-term work-experienced RNs. The research questions were:

- How do registered nurses self-report their job satisfaction, professional competence, and self-efficacy?
- Are there any differences in terms of self-reported job satisfaction, professional competence, and self-efficacy between newly qualified; medium-term work-experienced; and long-term work-experienced registered nurses?

## Methods

The reporting of this study was guided by The Strengthening the Reporting of Observational Studies in Epidemiology (STROBE) guidelines [26].

### Design and setting

This study was part of a larger overarching Swedish-Norwegian project. A multicenter cross-sectional survey study design was deemed suitable for describing features of a population in several locations at a single point in time, and useful for establishing evidence and a knowledge base for future studies and interventions [27]. The survey consisted of a selection of validated questionnaires: the Copenhagen Psychosocial Questionnaire (COPSOQ III) [28, 29], Nurse Professional Competence Scale Short Form (NPC Scale-SF) [30], and Occupational Self-Efficacy (OSE) scale [31].

The participating counties, one county in Sweden and one in Norway, are located in the northern parts of the countries and have similarities in terms of geography and demographics, being predominately rural areas [32]. Both Sweden and Norway have state-funded healthcare systems governed by a parliamentary system with three levels: state, region, and municipality. In Norway, the state (central government) owns, and finances specialized healthcare at a national level. Norway’s four regional health authorities control the provision of specialized health services. Healthcare in Sweden is decentralized, i.e., the responsibility lies with regional councils. The role of the central government is to establish principles and guidelines, and to set the political agenda for health and medical care. Primary healthcare serves as the foundation for healthcare in both countries. Municipalities oversee primary healthcare in Norway, regions manage primary healthcare in Sweden. Responsibility for elderly and home care lies with municipalities in both countries, governed by state laws.

There are 4614 employed RNs in the participating Norwegian county, in hospitals, primary health care, and municipalities [33]. Approximately 50% of all employees are members of Norwegian Union of Municipal and General Employees [34]. Most of the RNs are members of the Norwegian Nurses’ Organization, however, many of their members are also retired RNs, and RNs not actively working [35]. In Sweden, the participating county have 2929 RNs employed via the regional office in hospitals and primary health care [36]. However, not all actively working as RNs. The population in the present study worked in diverse hospital and prehospital care settings which included emergency department, anesthesia, operation room, intensive care unit, surgery and orthopedics, pediatrics, medicine, cardiology, neurology, oncology, geriatrics, labor and delivery, palliative care, and psychiatry. They could also work in primary healthcare, home care and nursing homes.

### Definitions

Defining a newly qualified nurse (NQN) can be nuanced, as there is no universally accepted definition. Previous studies have defined an NQN as an RN with up to one year of work experience [37–39] and up to two years of work experience [40]. In this study, an NQN is defined as an RN with up to 1.5 years of work experience. RNs with medium-term work experience are, in this study, defined as ones with 1.6–5 years of work experience; RNs with long-term work experience are defined as having more than 6 years of work experience. This categorization is based on previous research indicating that, after five years, there is an increased risk of stress, burnout, and intention to leave the profession. An RN experiencing stress early in their career can have long-term negative consequences such as burnout, and among those with five years of employment and symptoms of burnout, the prevalence of intention to leave the profession is 43% [2, 15].

### Data collection and participants

The study was conducted similarly in Sweden and Norway in September 2021, via a self-administered online survey. The target population was RNs working in healthcare units in the two participating counties. RNs working in patient care were included in the study, thus, those only working with non-clinical work were not eligible to participate, e.g., department managers and those with solely administrative work tasks. Of approx. 4000 RNs invited to participate, 1145 answered the survey – a response rate of 29%. We excluded respondents who were administrators (*n* = 8) from Swedish respondents, resulting in a total of 1137 participants in this study.

In Sweden, the surveys were distributed by email with a link to the questionnaire sent to all RNs employed in hospital care and primary healthcare through the regional office. In Norway, we gave instructions to only send the survey to actively working RNs and the questionnaires were distributed by email via the registers of the Norwegian Nurses’ Organization and the Norwegian Union of Municipal and General Employees. The survey was sent out in three rounds in both countries: an email; an email reminder after approximately three weeks, and a second email reminder after another three weeks.

The number of participants who worked in Sweden was 641 (56.4%), and in Norway 496 (43.6%), and most of the participants were women (84.7%). Age was stated in ranges; birth year of 1995 or later (age 26 years and under); 1980–1994 (age 27–41); 1965–1979 (age 42–56); 1964 or earlier (age 57 years and over). Most respondents were born between 1980 and 1994 (39.2%). Regarding type of employment, 94.1% of the participants were permanently employed, and 67.7% worked full-time. The majority had 6 or more years of experience working as an RN (72.8%).

Demographic characteristics for each work experience group are shown in Table 1.

**Table 1:** Demographic characteristics of the study participants, divided into work experience groups

	Work experience groups (Sweden)				Work experience groups (Norway)				Total (*n* = 1137)
	NQN n (%)	Medium-term n (%)	Long-term n (%)	*p*-value*	NQN n (%)	Medium-term n (%)	Long-term n (%)	*p*-value*	n (%)
Participants	70 (10.9)	127 (19.8)	444 (69.3)		40 (8.1)	72 (14.5)	384 (77.4)		1137 (100)
Gender				0.323				0.187	
Female	59 (84.3)	101 (79.5)	367 (82.7)		38 (95.0)	63 (87.5)	335 (87.2)		963 (84.7)
Male	11 (15.7)	24 (18.9)	76 (17.1)		1 (2.5)	9 (12.5)	47 (12.2)		168 (14.8)
Other/non-binary	-	2 (1.6)	1 (0.2)		1 (2.5)	-	2 (0.5)		6 (0.5)
Birth year				**< 0.001**				**< 0.001**	
1964 or earlier	-	1 (0.8)	94 (21.2)		1 (2.5)	1 (1.4)	97 (25.3)		194 (17.1)
1965–1979	1 (1.4)	11 (8.7)	190 (42.8)		3 (7.5)	7 (9.7)	163 (42.4)		375 (33.0)
1980–1994	25 (35.7)	91 (71.7)	159 (35.8)		10 (25.0)	38 (52.8)	123 (32.0)		446 (39.2)
1995 or later	44 (62.9)	24 (18.9)	1 (0.2)		26 (65.0)	26 (36.1)	1 (0.3)		122 (10.7)
Education				**< 0.001**				**< 0.001**	
Specialist and/or master’s degree	-	27 (21.3)	249 (56.1)		4 (10.0)	21 (29.2)	240 (62.5)		541 (47.6)
Other postgraduate education^1^	4 (5.7)	15 (11.8)	54 (12.1)		9 (22.5)	14 (19.4)	50 (13.0)		146 (12.8)
No postgraduate education	66 (94.3)	85 (66.9)	141 (31.8)		27 (67.5)	37 (51.4)	94 (24.5)		450 (39.6)
Area of work				**< 0.001**				**< 0.001**	
Hospital/prehospital care^2^	64 (91.4)	119 (93.7)	414 (93.2)		19 (47.5)	22 (30.5)	66 (17.2)		704 (61.9)
Primary healthcare	-	7 (5.5)	22 (5.0)		2 (5.0)	-	17 (4.4)		48 (4.2)
Municipality/nursing home	3 (4.3)	-	-		14 (35.0)	30 (41.7)	145 (37.8)		192 (16.9)
Other/not specified^3^	3 (4.3)	1 (0.8)	8 (1.8)		5 (12.5)	20 (27.8)	156 (40.6)		193 (17.0)
Employment status^4^				**< 0.001**				0.335	
Full-time	62 (88.6)	100 (78.7)	275 (61.9)		24 (60.0)	46 (63.9)	263 (68.5)		770 (67.7)
Part-time	8 (11.4)	27 (21.3)	168 (37.8)		16 (40.0)	26 (36.1)	121 (31.5)		366 (32.2)
Type of contract				**< 0.001**				**< 0.001**	
Permanent	55 (78.6)	119 (93.7)	441 (99.3)		27 (67.5)	61 (84.7)	367 (95.6)		1070 (94.1)
Temporary	15 (21.4)	8 (6.3)	3 (0.7)		13 (32.5)	11 (15.3)	17 (4.4)		67 (5.9)

^1^ “Other postgraduate education” refers to courses and educational programs pursued after obtaining a bachelor’s degree in nursing. This includes supplementary education and courses in supervision and preceptorship. ^2^ In this study “Hospital/prehospital care” extends to advanced home care, distinguishing it from the care provided in nursing homes or municipal settings. ^3^ “Other/not specified” in “Area of work” could be private healthcare, outpatient care. ^4^ Missing (*n* = 1) from Swedish data. *Pearson chi-square test, statistically significant *p*-values are bolded.

### Questionnaires and outcome variables

The survey included background questions, and 46 questions covering self-reported job satisfaction, professional competence, and self-efficacy in Swedish and Norwegian, drawing from questionnaires validated in the Scandinavian context [28–30, 41].

The Copenhagen Psychosocial Questionnaire (COPSOQ III) [28, 29], recognized as a risk-assessment tool by the World Health Organization [29], covers the working environment, conflict, offensive behavior, and health and welfare. For job satisfaction, a five-item dimension was included, with response options on a five-point Likert scale, scored as 0 (very unsatisfied), 25 (unsatisfied), 50 (neither/nor), 75 (satisfied), and 100 (very satisfied). In accordance with COPSOQ III scoring instructions [28], a median scale value was calculated with range 0–100. Also, a median score of the mandatory core item “Your job as a whole, everything taken into consideration” between 0 and 100 was calculated. Internal consistency of the scale was examined, with Cronbach’s alpha coefficient of 0.773.

The Nurse Professional Competence (NPC) Short Form (SF) scale [30], which is based on Benner’s framework [19], consists of 35 items across six theoretical factors: Nursing care, Value-based nursing care, Medical and technical care, Care pedagogics, Documentation and administration of nursing care, and Development, leadership, and organization of nursing care. The response options were on a seven-point Likert scale, ranging from 1 (very low) to 7 (very high). The scores of the items in a factor were summarized, divided by the highest possible score in that factor, and multiplied by 100, giving a 1–100 scale for each factor, in accordance to scoring instructions [30, 42, 43]. When the surveys were collected, one item from the NPC-SF scale was accidentally omitted from 42 questionnaires in Sweden: “Inform and educate groups of patients and relatives”, from the “Care pedagogics” factor. This item was consequently removed from the analysis, resulting in a total of 34 items in all questionnaires. The internal consistency of the scale was examined, resulting in a Cronbach’s alpha coefficient of 0.951.

Self-efficacy was assessed using the Occupational Self-efficacy (OSE) Scale [31, 41] in Sweden, and since the OSE scale was not validated in Norway, we instead used the self-efficacy dimension from COPSOQ III in Norway. The OSE Scale includes six items on a six-point Likert scale, ranging from 1 (not at all true) to 6 (completely true). High values reflect high reported occupational self-efficacy, with a minimum total score of 6 and maximum total score of 36. The OSE scale has shown structural and construct validity [31]. When collecting the surveys, one item from the OSE Scale was accidentally omitted from 42 questionnaires: “My studies have prepared me for my current position”. These are counted as missing in the analysis. Internal consistency of the scale was examined, with Cronbach’s alpha coefficient of 0.820. The COPSOQ III self-efficacy dimension used in Norway has six items on a four-point Likert scale, scored 0 (does not fit), 33 (fits a little), 67 (fits quite well), 100 (fits perfectly) [44]. In accordance with COPSOQ III scoring instructions [28], a median scale value was calculated with range 0–100. Internal consistency was examined, with Cronbach’s alpha coefficient of 0.785.

### Statistics

Demographic data and responses to scale items were analyzed with descriptive and comparative statistics using IBM® SPSS® Statistics version 29 (IBM Corp, Armonk, NY). Demographic data are presented with counts and percentages, and the participants were categorized into three groups according to their work experience. This categorization was based on previous research, as described in the background of this paper. The participants were categorized as “NQN” (≤ 1.5 years of work experience), “medium-term” (1.6 to 5 years of work experience), and “long-term” (≥ 6 years of work experience). A chi-square test was used to compare differences in demographic variables across the groups.

For our outcome variables, we merged responses from Sweden and Norway since the results were similar for both countries, separate analyses were therefore not motivated. We did not present results from multivariable analyses as the results were similar across variables such as nationality, age group, education, and area of work, which could potentially confound our findings. However, as there are differences in scales and outcomes in self-efficacy, the results on this variable are presented separately for Sweden and Norway, in order to accurately reflect the results.

Questionnaire scale scores was statistically treated in accordance with each instrument’s instructions regarding the analysis procedure. The distributions of variables job satisfaction and self-efficacy (Norwegian) were skewed, therefore descriptive and comparative statistics for these outcome variables are presented with medians and quartiles. The differences between the work experience groups with regard to these variables were analyzed using the non-parametric Kruskal-Wallis test as results from the variable was too skewed to make comparisons between means meaningful.

The scales for professional competence and self-efficacy (Swedish) are presented with mean, standard deviation, and confidence intervals (95%). Differences between the work experience groups with regard to these variables were analyzed using a one-way between-groups analysis of variance (ANOVA).

When the comparative tests revealed statistically significant differences, a post-hoc test, Bonferroni correction, was used for multiple comparisons. A *p*-value of < 0.05 was regarded as statistically significant in all analyses. The effect sizes were calculated using eta squared.

## Results

### Job satisfaction

The median scale score for job satisfaction in RNs with medium-term work experience was significantly lower (Md = 51.0) than that of NQNs (Md = 58.2, *p* = 0.011) and long-term work-experienced RNs (Md = 59.1, *p* < 0.001). The scores of NQNs did not differ significantly from those of long-term work-experienced RNs. On the job satisfaction core item “Your job as a whole, everything taken into consideration” the same pattern was seen; RNs with medium-term work experience reported lower job satisfaction than the other two groups (*p* = 0.022, < 0.001, respectively) (Table 2).

**Table 2:** Self-reported scores on job satisfaction, professional competence, and self-efficacy, per work experience group, and comparisons between groups

	NQN (1)			Medium-term (2)			Long-term (3)							
Job satisfaction	n	Md	Q1–Q3	n	Md	Q1–Q3	n	Md	Q1–Q3	p*	η^2^	p (1–2)	p (1–3)	p (2–3)
Median scale score^1^	107	58.2	45–65	197	51.0	40–60	815	59.1	45–70	**< 0.001**	0.019	**0.011**	0.329	**< 0.001**
Core item score^1^	109	70.2	50–75	199	64.1	50–75	825	70.8	50–75	**< 0.001**	0.014	**0.022**	1.000	**< 0.001**
**Professional competence** ^**2**^	**n**	**M (SD)**	**CI 95%**	**n**	**M (SD)**	**CI 95%**	**n**	**M (SD)**	**CI 95%**	p**	**η** ^**2**^	**p** **(1–2)**	**p** **(1–3)**	**p** **(2–3)**
Nursing care	110	83.0 (9.9)	81.1–84.9	199	81.7 (10.4)	80.3–83.1	828	84.0 (10.9)	83.2–84.7	**0.026**	0.006	0.559	0.660	**0.021**
Value-based nursing care	110	84.0 (9.7)	82.2–85.9	199	84.1 (10.8)	82.6–85.6	828	85.9 (10.6)	85.1–86.6	**0.037**	0.006	1.000	0.201	0.079
Medical-technical care	110	79.9 (10.4)	77.9–81.8	199	83.0 (10.8)	81.5–84.5	828	85.9 (11.4)	84.2–85.8	**< 0.001**	0.020	**0.049**	**< 0.001**	0.059
Care pedagogics	110	78.8 (11.7)	76.4–80.9	199	78.3 (11.7)	76.7–79.9	828	80.5 (13.9)	79.5–81.4	0.068^a^	0.005	-	-	-
Documentation and administration	110	75.4 (11.0)	73.4–77.5	199	77.2 (11.1)	75.6–78.7	828	79.0 (10.9)	78.2–79.7	**0.002**	0.011	0.377	**0.004**	0.091
Development and leadership	110	75.5 (11.5)	73.3–77.6	199	75.6 (11.3)	74.0–77.2	828	79.3 (11.8)	78.5–80.1	**< 0.001**	0.020	0.993	**0.003**	**< 0.001**
**Self-efficacy (Sweden)**	**n**	**M (SD)**	**CI 95%**	**n**	**M (SD)**	**CI 95%**	**n**	**M (SD)**	**CI 95%**	p**	**η** ^**2**^	**p** **(1–2)**	**p** **(1–3)**	**p** **(2–3)**
Total scale score^3^	26	24.5 (4.9)	22.5–26.5	125	25.4 (3.9)	24.7–26.1	433	28.1 (4.4)	27.7–28.6	**< 0.001**	0.081	0.623	**< 0.001**	**< 0.001**
**Self-efficacy (Norway)**	**n**	**Md**	**Q1–Q3**	**n**	**Md**	**Q1–Q3**	**n**	**Md**	**Q1–Q3**	p*	**η** ^**2**^	**p** **(1–2)**	**p** **(1–3)**	**p** **(2–3)**
Median scale score^1^	40	73.6	60.1–87	72	73.6	60–80.4	384	73.6	60.2–86.6	0.193^a^	0.002	-	-	-

^1^ Min 0 – max 100. ^2^ Min 1 – max 100 (5–8 items per factor). ^3^ Min 6 – max 36 (6 items). Md = Median, Q1–Q3 = Lower and upper quartile. M = mean, SD = standard deviation, CI = confidence interval. η2 = Eta squared (effect size). *Kruskal-Wallis test with post-hoc Bonferroni for multiple comparisons. **ANOVA test with post-hoc Bonferroni for multiple comparisons. Statistically significant *p*-values are bolded. ^a^ Group-wise comparisons were not made because there was no significance.

The lowest rating on an individual item in the job satisfaction scale was for the item “Your salary” (not shown in table); the median value for NQNs was 21.6, for medium-term work-experienced RNs 20.2, and for long-term work-experienced RNs 30.7. There were statistically significant differences for this item across the groups (*p* < 0.001), between NQNs and long-term work-experienced RNs (*p* = 0.047), and between medium-term and long-term work-experienced RNs (*p* < 0.001).

### Professional competence

Professional competence was reported as highest among RNs with long-term work experience (M = 79.0-85.9). When comparing scores on each factor, there were statistically significant differences in each factor across the three different work experience groups, with the exception of the factors “Care pedagogics” and “Value-based nursing care” (Table 2).

Statistically significant differences were found between RNs with long-term work experience and NQNs in factors “Medical-technical care” (*p* < 0.001), “Documentation and administration” (*p* = 0.004) and “Development and leadership” (*p* = 0.003). Statistically significant differences were also found between RNs with long-term work experience and RNs with medium-term work experience in factors “Nursing care” (*p* = 0.021) and “Development and leadership” (*p* < 0.001). There were no statistically significant differences between NQNs and the other two groups in “Nursing care” (Table 2).

NQNs reported their competence highest for the factor “Value-based nursing care”, and lowest for the factor “Documentation and administration”. Medium-term work-experienced RNs reported their competence highest for the factor “Value-based nursing care” and lowest for the factor “Development and leadership”. Long-term work-experienced RNs reported their competence highest for the factors “Value-based nursing care” and “Medical-technical care”, and lowest for the factor “Documentation and administration” (Table 2).

### Self-efficacy

Among Swedish RNs, self-efficacy (occupational) was reported as highest among RNs with long-term work experience (M = 28.1). There were statistically significant differences (*p* < 0.001) between RNs with long-term work experience and medium-term work experience, as well as between RNs with long-term work experience and NQNs. The scores of NQNs did not differ significantly from those of medium-term work-experienced RNs (Table 2).

The individual item with the lowest reported scores across all work experience groups of Swedish RNs was “My studies have prepared me for my current position” (not shown in table), which had a mean of 3.1 (SD 1.3). Among Norwegian RNs, there were no statistically significant differences in reported self-efficacy between the work experience groups (Table 2).

## Discussion

In this study, the aim was to investigate self-reported job satisfaction, professional competence, and self-efficacy of RNs in one northern county in Sweden and one in Norway. Analysis revealed notable variations in job satisfaction, professional competence, and self-efficacy across different work-experienced groups. The results show that job satisfaction was reported as lowest among RNs with medium-term work experience; NQNs and RNs with long-term work experience reported higher job satisfaction. The results also show that RNs with long-term work experience reported higher professional competence and self-efficacy compared to NQNs and medium-term work-experienced RNs.

Regarding job satisfaction, we have not found any studies with similar comparisons on this scale between work experience groups. Reference values for RNs on job satisfaction in COPSOQ III are mean 64.4 (out of 100) for all work areas and mean 68 (out of 100) for RNs [28]. Thus, there are difficulties in comparing our results in job satisfaction to previous studies. For all groups in the present study, overall median scale score was lower than the median score for core item “Your job as a whole, everything taken into consideration”, indicating that even though there are items, such as “salary”, that lower their overall median score, they still rate that that they are satisfied with their job as a whole.

Interestingly, within the job satisfaction scale, all groups reported lowest satisfaction with their salary. This result diverges from a US study that highlighted that salary is overshadowed by factors such as work environment and staffing [45]. Also, a Finnish study found that non-financial rewards are valued higher than financial rewards (i.e., salary) for RNs job satisfaction [46]. However, a recent Swedish study confirmed that recognition, including salary, is a crucial motivator for experienced RNs, and influences their intention to stay in the profession [47]. An interpretation is that salary is more important for RNs now than reported in the previous studies [45, 46]. Another interpretation is that the present study was conducted during the COVID-19 pandemic, with increased sick leave, salary might be considered more important.

In terms of professional competence, RNs with long-term work experience reported significantly higher competence than NQNs and RNs with medium-term work experience. This finding contrasts with a prior Swedish study that examined NQNs upon graduation, wherein they self-reported their professional competence equally high or higher than RNs with more work experience [48]. Notably, the factor “Nursing care” showed no statistically significant discrepancies between NQNs and the other groups in our current investigation. Furthermore, RNs surveyed in this study indicated lower professional competence (mean factor scores ranging from 75.4 to 85.9 across all groups) compared to RNs in a study conducted in Korea (mean factor scores ranging from 80.4 to 87.1) [49].

In a cross-sectional study in Poland, NQNs reporting on their professional competence scored the factors “Nursing care” and “Value-based nursing care” the highest, and “Development and leadership” the lowest [50]. The results of the present study confirm this: all the surveyed RNs reported the highest professional competence in relation to the same factor – “Value-based nursing care” – regardless of their work experience. Interestingly, RNs with long-term work experience surveyed in this study reported their competence to be lowest in relation to the “Documentation and administration” factor, which the NQNs also reported as their lowest factor. RNs with medium-term work experience reported their competence to be lowest in the “Development and leadership” factor. This implies that these factors need to be targeted for educational and developmental measures in the workplace, since they are reported as the lowest even among those with the most work experience.

The reported self-efficacy of the Swedish RNs was higher than that of RNs in a Chinese study, where long-term work-experienced RNs had a mean self-efficacy rating of 24.8 (mean 28.1 in our study), and medium-term work-experienced RNs and NQNs had mean of 22.1 [51] (mean 25.4 and 24.5, respectively, in our study). While we have not conducted a statistical analysis to determine significance, it is noteworthy that the RNs surveyed in our study reported higher levels of self-efficacy as compared to those in the study mentioned in the previous sentence. It is also worth noting again that the present study was conducted during the COVID-19 pandemic. Numerous studies investigated self-efficacy during the pandemic, although applying different self-efficacy scales but, for example, studies in Italy [52] and Indonesia [53] concluded that self-efficacy was low in RNs at this time. However, the present study confirms the results of a previous study in Poland, which found that self-efficacy was reported as higher among RNs with long-term work experience during the pandemic, compared to RNs with less work experience [54].

Notably, the lowest self-efficacy score among the Swedish RNs across all work experience groups in our study was for the item “My studies have prepared me for my current position”. This confirms the findings of a study of NQNs in Iran, who described insufficient knowledge acquisition during their education affecting their self-confidence as RNs [55]. This emphasizes the need for ongoing professional development and continuing learning and support in the workplace, as well as further development of nursing education to better match the demands of today’s nursing practice.

In the present study, we found that in the group of long-term work experienced RNs, professional competence and self-efficacy were reported as higher (with the exception of self-efficacy in the Norwegian data) than in groups with medium-term work experience and NQNs. Among Norwegian participants, there were no differences in self-efficacy between work experience groups. This result is confirmatory to the results of a recent Italian study, which found no correlation between work years and reported self-efficacy [23]. However, another study concluded that RNs who reported lower levels of competence were also less likely to report high self-efficacy and job satisfaction. Conversely, those RNs who reported their competence as high tended to report higher self-efficacy and job satisfaction [56]. The results of the present study contradict this. RNs with medium-term work experience reported lower job satisfaction than NQNs and long-term work-experienced RNs. NQNs reported similar scores of job satisfaction as long-term work-experienced RNs, even though their self-efficacy and professional scores were lower than long-term work-experienced RNs. This divergence highlights the complexity of factors that influence job satisfaction. Further exploration of these multifaceted dynamics could provide valuable insights for nursing practice and policy.

### Conclusions and implications

By addressing the challenges inherent in conducting multi-center research and leveraging a diverse participant sample, this study provides valuable insights to inform policy and practice in supporting and retaining RNs. These results underscore the importance of targeted support mechanisms, particularly during the transition from newly qualified to experienced phases, to enhance job satisfaction. This, in turn, might cultivate a resilient and sustainable nursing workforce, which is essential for ensuring safe and effective patient care. Interestingly, across all work experience groups, “Value-based nursing care” was the highest-scoring item on the professional competence scale. This raises the question of which factors are essential to target for professional development strategies. Furthermore, ongoing professional development initiatives tailored to address areas of perceived competence deficits are essential for nurturing a stable working life in Nordic healthcare organizations.

The discrepancy in reported job satisfaction between medium-term work-experienced RNs compared to NQNs and long-term work-experienced RNs needs further investigation to identify the underlying factors and develop targeted interventions to address them effectively. Continued research in this area is imperative in terms of the development of evidence-based strategies to promote the well-being and retention of RNs.

### Methodological strengths and limitations

This study has several strengths. The sample size was large, which resulted in strong and reliable results and increased our ability to make conclusions regarding the population [57]. However, we had a low response rate, which could decrease the generalizability of our results. Data-collection challenges often arise in the context of research involving RNs, who have limited availability and time to spare. Survey response rates of less than 50% are common for survey studies involving RNs [58]. Exact numbers of distributed surveys in our study are not available, since they were distributed to all actively working RNs, we estimated the numbers of distributed surveys.

The multicenter design, with diverse orientations and perspectives, along with the fact that the study was conducted in two counties in different countries, resulted in diverse population coverage and increased generalizability [59]. This highly important topic of the working conditions of RNs makes the study worthwhile, although it was challenging to conduct (possibly due to the impact of the pandemic).

During this study we faced several challenges. With surveys, there is always the risk of non-response bias, and a systematic difference between responders and non-responders [27]. The approach of recruiting participants via surveys distributed through the regional office in Sweden, Norwegian Nurses’ Organization, and Norwegian Union of Municipal and General Employees in Norway could have influenced the respondents. However, these recruitment methods facilitated the inclusion of participants spanning diverse ages, genders, and organizational contexts, across municipalities and counties. Establishing a diverse and representative sample decreases the risk of non-response bias, which is only a detriment when respondents differ from non-respondents in substantial ways [58].

Since the survey was anonymous, we sent reminders to every participant. This means that there is a possibility that one or more people responded to the survey twice. However, it was clearly stated in the reminders that only those who had not previously completed the survey were to respond, emphasizing that it was a reminder. In the survey, we asked the participants in which month of which year they graduated, and some participants stated only the year; in these instances, we calculated the time from the survey date, September 2021. This could be a methodological limitation in terms of the accuracy of the dates.

The development of the survey began with the same questions and design in both countries. However, the ethical approvals were not identical; in Norway we were not approved to collect the exact ages of the participants, thus, age is stated in ranges. Regarding self-efficacy scales, we used two different scales related to the fact that the OSE scale was not validated in Norway, which is a methodological limitation of this study. The scales for professional competence and job satisfaction where the same for both countries. In summary, the benefits of reaching a diverse population across two different countries outweigh the drawbacks of this multicenter study.

## Data Availability

The datasets used and/or analyzed during the current study are available from the corresponding author on reasonable request.
